# 4-Eth­oxy­anilinium bromide

**DOI:** 10.1107/S1600536810048713

**Published:** 2010-11-27

**Authors:** Wen-zhe Wang

**Affiliations:** a College of Food Engineering, RiZhao Polytechnic, RiZhao 276826, People’s Republic of China

## Abstract

The title compound, C_8_H_12_NO^+^·Br^−^, is built up from roughly planar (r.m.s. deviation for the non-H atoms = 0.062 Å) protonated 4-eth­oxy­anilimium cations and Br^−^ anions. In the crystal, the cations and anions are linked by N—H⋯Br and N—H⋯(Br,Br) hydrogen bonds, generating (100) sheets. Very weak C—H⋯π inter­actions may also help to stabilize the crystal structure.

## Related literature

For a related structure containing the same cation, see: Fu (2009 ▶).
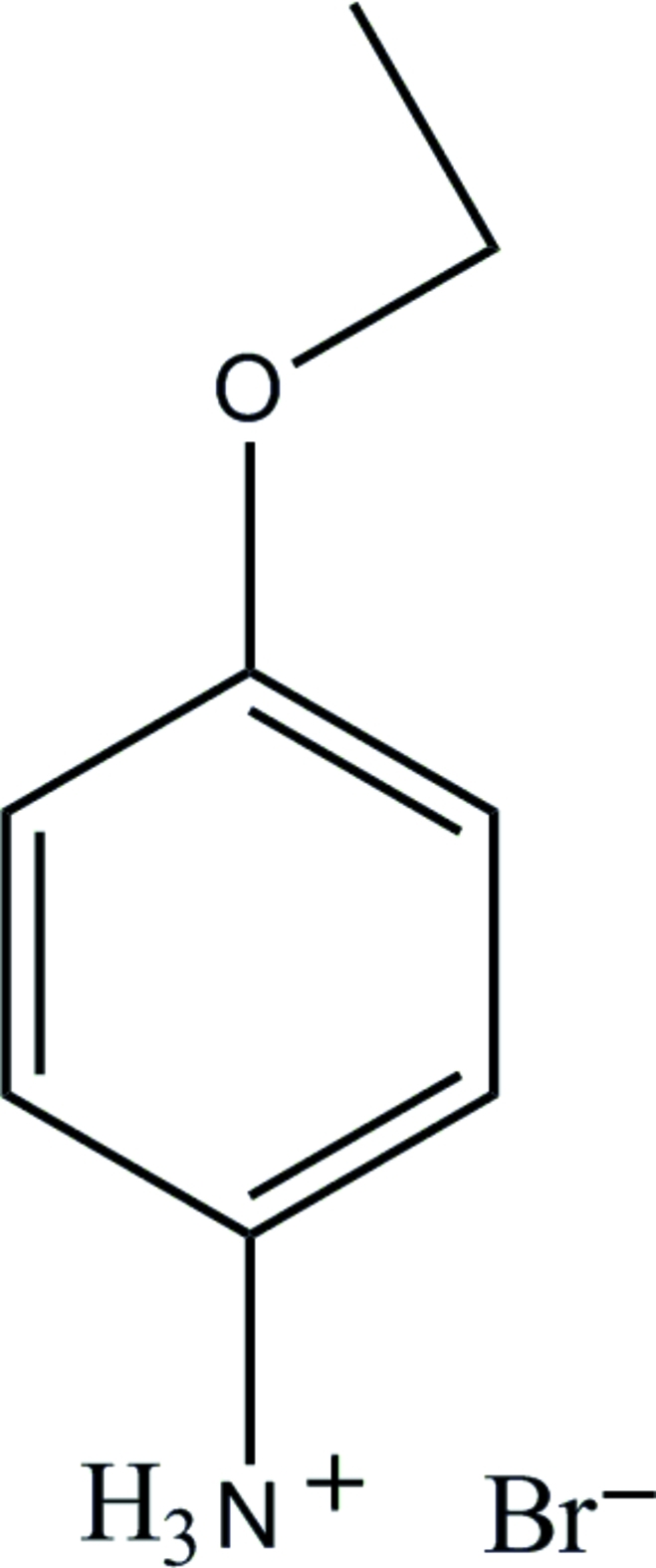


## Experimental

### 

#### Crystal data

C_8_H_12_NO^+^·Br^−^
*M* *_r_* = 218.09
Monoclinic, 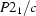

*a* = 11.842 (2) Å
*b* = 6.5263 (13) Å
*c* = 12.488 (3) Å
β = 96.44 (3)°
*V* = 959.0 (3) Å^3^
*Z* = 4
Mo *K*α radiation
μ = 4.23 mm^−1^
*T* = 298 K
0.40 × 0.30 × 0.20 mm

#### Data collection

Rigaku SCXmini diffractometer
Absorption correction: multi-scan (*CrystalClear*; Rigaku, 2005 ▶) *T*_min_ = 0.237, *T*_max_ = 0.429
9286 measured reflections
2200 independent reflections
1579 reflections with *I* > 2σ(*I*)
*R*_int_ = 0.073

#### Refinement

*R*[*F* ^2^ > 2σ(*F* ^2^)] = 0.061
*wR*(*F* ^2^) = 0.173
*S* = 1.11
2200 reflections
100 parameters
H-atom parameters constrained
Δρ_max_ = 1.55 e Å^−3^
Δρ_min_ = −0.42 e Å^−3^

### 

Data collection: *CrystalClear* (Rigaku, 2005 ▶); cell refinement: *CrystalClear*; data reduction: *CrystalClear*; program(s) used to solve structure: *SHELXS97* (Sheldrick, 2008 ▶); program(s) used to refine structure: *SHELXL97* (Sheldrick, 2008 ▶); molecular graphics: *SHELXTL* (Sheldrick, 2008 ▶); software used to prepare material for publication: *SHELXTL*.

## Supplementary Material

Crystal structure: contains datablocks I, global. DOI: 10.1107/S1600536810048713/hb5753sup1.cif

Structure factors: contains datablocks I. DOI: 10.1107/S1600536810048713/hb5753Isup2.hkl

Additional supplementary materials:  crystallographic information; 3D view; checkCIF report

## Figures and Tables

**Table 1:** Hydrogen-bond geometry (Å, °) *Cg*1 is the centroid of the benzene ring.

*D*—H⋯*A*	*D*—H	H⋯*A*	*D*⋯*A*	*D*—H⋯*A*
N1—H1*B*⋯Br1	0.89	2.78	3.368 (4)	125
N1—H1*B*⋯Br1^i^	0.89	2.76	3.324 (5)	122
N1—H1*C*⋯Br1^ii^	0.89	2.56	3.375 (4)	153
N1—H1*D*⋯Br1^iii^	0.89	2.51	3.348 (5)	158
C7—H7*A*⋯*Cg*1^iv^	0.97	3.01	3.674 (8)	127
C8—H8*B*⋯*Cg*1^v^	0.96	2.96	3.677 (8)	132

Symmetry codes: (i) 
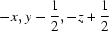
; (ii) 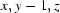; (iii) 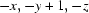; (iv) 
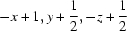
; (v) 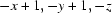.

**Fig. 1.**
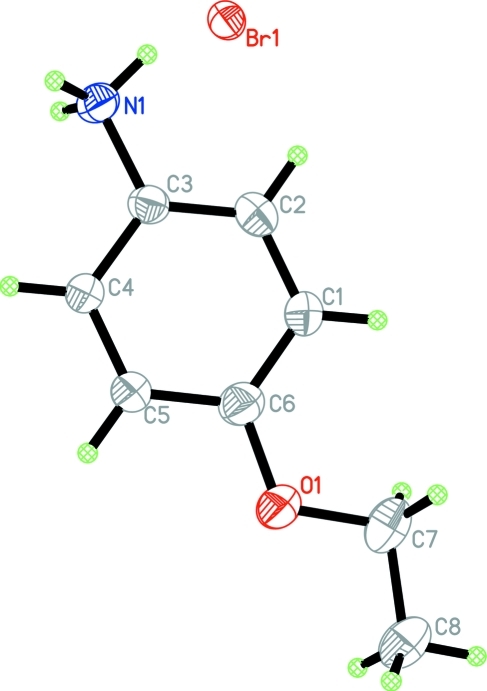
The molecular structure of the title compound, with displacement ellipsoids drawn at the 30% probability level.

**Fig. 2.**
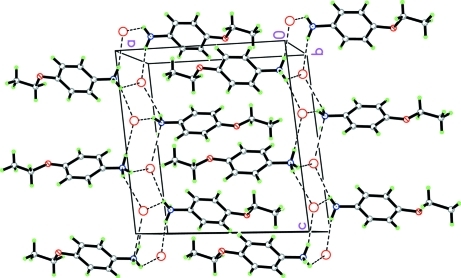
A view of the packing of the title compound, stacking along the c axis. Dashed lines indicate hydrogen bonds.

## supplementary crystallographic information

### Comment

The crystal structure of 4-ethoxyanilinium together with perchlorate is known (Fu, 2009).

The asymmetric unit of the title compound consists of an almost planar protonated 4-ethoxyanilimium cation with the mean deviation of 0.0618 A from the plane formed by its non-hydrogen atoms and a Br^-^ anion (Fig.1). The N—H···Br hydrogen bonding with the N—Br distance from 3.324 (5)Å to 3.375 (4) Å, make great contribution to the stability of the crystal structure and link the cations and anions into chains along *b* axis. The C—H···π interactions with the C···*Cg*1 distances of C7—H7A···*Cg*1 3.674 (8)Å and C8—H8B···*Cg*1 3.677 (8) Å, respectively, (*Cg*1 is the centroid of benzene ring) also help stable crystal structure.

### Experimental

Colorless prisms of the title compound were obtained by slow evaporation at room temperature of an ethanol solution of equimolar amounts of 4-ethoxybenzenamine and hydrobromic acid (47% *w*/*w*).

### Refinement

Positional parameters of all the H atoms for C/N atoms were calculated geometrically and were allowed to ride on the C atoms to which they are bonded, with *U*_iso_(H) = 1.2Ueq(C), *U*_iso_(H) = 1.5Ueq(C) for methyl group and *U*_iso_(H) = 1.5Ueq(N).

### Crystal data

**Table:**

C_8_H_12_NO^+^·Br^−^	*F*(000) = 440
*M**_r_* = 218.09	*D*_x_ = 1.510 Mg m^−^^3^
Monoclinic, *P*2_1_/*c*	Mo *K*α radiation, λ = 0.71073 Å
Hall symbol: -P 2ybc	Cell parameters from 3858 reflections
*a* = 11.842 (2) Å	θ = 3.1–27.7°
*b* = 6.5263 (13) Å	µ = 4.23 mm^−^^1^
*c* = 12.488 (3) Å	*T* = 298 K
β = 96.44 (3)°	Prism, colourless
*V* = 959.0 (3) Å^3^	0.40 × 0.30 × 0.20 mm
*Z* = 4	

### Data collection

**Table:**

Rigaku SCXmini diffractometer	2200 independent reflections
Radiation source: fine-focus sealed tube	1579 reflections with *I* > 2σ(*I*)
graphite	*R*_int_ = 0.073
Detector resolution: 13.6612 pixels mm^-1^	θ_max_ = 27.5°, θ_min_ = 3.3°
ω scans	*h* = −15→15
Absorption correction: multi-scan (*CrystalClear*; Rigaku, 2005)	*k* = −8→8
*T*_min_ = 0.237, *T*_max_ = 0.429	*l* = −16→16
9286 measured reflections	

### Refinement

**Table:**

Refinement on *F*^2^	Primary atom site location: structure-invariant direct methods
Least-squares matrix: full	Secondary atom site location: difference Fourier map
*R*[*F*^2^ > 2σ(*F*^2^)] = 0.061	Hydrogen site location: inferred from neighbouring sites
*wR*(*F*^2^) = 0.173	H-atom parameters constrained
*S* = 1.11	*w* = 1/[σ^2^(*F*_o_^2^) + (0.0748*P*)^2^ + 1.0824*P*] where *P* = (*F*_o_^2^ + 2*F*_c_^2^)/3
2200 reflections	(Δ/σ)_max_ < 0.001
100 parameters	Δρ_max_ = 1.55 e Å^−^^3^
0 restraints	Δρ_min_ = −0.42 e Å^−^^3^

### Special details

**Table:**

Geometry. All e.s.d.'s (except the e.s.d. in the dihedral angle between two l.s. planes) are estimated using the full covariance matrix. The cell e.s.d.'s are taken into account individually in the estimation of e.s.d.'s in distances, angles and torsion angles; correlations between e.s.d.'s in cell parameters are only used when they are defined by crystal symmetry. An approximate (isotropic) treatment of cell e.s.d.'s is used for estimating e.s.d.'s involving l.s. planes.
Refinement. Refinement of *F*^2^ against ALL reflections. The weighted *R*-factor *wR* and goodness of fit *S* are based on *F*^2^, conventional *R*-factors *R* are based on *F*, with *F* set to zero for negative *F*^2^. The threshold expression of *F*^2^ > σ(*F*^2^) is used only for calculating *R*-factors(gt) *etc*. and is not relevant to the choice of reflections for refinement. *R*-factors based on *F*^2^ are statistically about twice as large as those based on *F*, and *R*- factors based on ALL data will be even larger.

### Fractional atomic coordinates and isotropic or equivalent isotropic displacement parameters (Å^2^)

**Table:**

	*x*	*y*	*z*	*U*_iso_*/*U*_eq_	
Br1	0.01352 (5)	0.76484 (8)	0.12594 (4)	0.0493 (3)	
O1	0.5504 (4)	0.2634 (6)	0.0985 (5)	0.0665 (14)	
N1	0.0856 (4)	0.2654 (7)	0.1354 (4)	0.0514 (12)	
H1B	0.0671	0.3613	0.1810	0.077*	
H1C	0.0632	0.1434	0.1568	0.077*	
H1D	0.0516	0.2921	0.0697	0.077*	
C6	0.4385 (6)	0.2722 (9)	0.1145 (6)	0.0575 (16)	
C4	0.2549 (5)	0.1252 (9)	0.0666 (5)	0.0551 (15)	
H4A	0.2087	0.0301	0.0274	0.066*	
C3	0.2087 (5)	0.2643 (7)	0.1331 (5)	0.0458 (13)	
C5	0.3688 (5)	0.1288 (10)	0.0589 (5)	0.0608 (17)	
H5A	0.4001	0.0332	0.0156	0.073*	
C1	0.3918 (6)	0.4084 (9)	0.1824 (6)	0.0640 (18)	
H1A	0.4380	0.5023	0.2225	0.077*	
C8	0.7385 (6)	0.3844 (11)	0.1062 (6)	0.073 (2)	
H8A	0.7906	0.4880	0.1355	0.109*	
H8B	0.7315	0.3907	0.0289	0.109*	
H8C	0.7665	0.2519	0.1296	0.109*	
C2	0.2756 (6)	0.4042 (10)	0.1902 (5)	0.0630 (17)	
H2A	0.2438	0.4972	0.2346	0.076*	
C7	0.6253 (6)	0.4193 (11)	0.1441 (6)	0.075 (2)	
H7A	0.6314	0.4131	0.2221	0.089*	
H7B	0.5965	0.5534	0.1213	0.089*	

### Atomic displacement parameters (Å^2^)

**Table:**

	*U*^11^	*U*^22^	*U*^33^	*U*^12^	*U*^13^	*U*^23^
Br1	0.0600 (4)	0.0431 (4)	0.0460 (4)	−0.0070 (3)	0.0119 (3)	−0.0002 (2)
O1	0.046 (2)	0.064 (3)	0.090 (4)	−0.0075 (19)	0.010 (2)	−0.017 (2)
N1	0.043 (3)	0.061 (3)	0.050 (3)	−0.007 (2)	0.009 (2)	0.000 (2)
C6	0.050 (4)	0.056 (4)	0.068 (4)	−0.004 (3)	0.012 (3)	−0.002 (3)
C4	0.045 (3)	0.055 (3)	0.067 (4)	−0.011 (3)	0.016 (3)	−0.019 (3)
C3	0.045 (3)	0.044 (3)	0.049 (3)	0.002 (2)	0.010 (2)	−0.002 (2)
C5	0.048 (4)	0.059 (4)	0.077 (5)	−0.007 (3)	0.016 (3)	−0.025 (3)
C1	0.052 (4)	0.064 (4)	0.077 (5)	−0.010 (3)	0.009 (3)	−0.027 (3)
C8	0.053 (4)	0.083 (5)	0.082 (5)	−0.014 (4)	0.006 (4)	0.013 (4)
C2	0.061 (4)	0.061 (4)	0.068 (4)	0.002 (3)	0.011 (3)	−0.021 (3)
C7	0.060 (5)	0.074 (5)	0.088 (6)	−0.021 (4)	−0.001 (4)	0.002 (4)

### Geometric parameters (Å, °)

**Table:**

O1—C6	1.363 (9)	C3—C2	1.358 (8)
O1—C7	1.426 (7)	C5—H5A	0.9300
N1—C3	1.461 (8)	C1—C2	1.390 (9)
N1—H1B	0.8900	C1—H1A	0.9300
N1—H1C	0.8900	C8—C7	1.489 (9)
N1—H1D	0.8900	C8—H8A	0.9600
C6—C5	1.382 (9)	C8—H8B	0.9600
C6—C1	1.385 (9)	C8—H8C	0.9600
C4—C5	1.362 (8)	C2—H2A	0.9300
C4—C3	1.384 (7)	C7—H7A	0.9700
C4—H4A	0.9300	C7—H7B	0.9700
			
C6—O1—C7	118.9 (5)	C6—C1—C2	119.8 (6)
C3—N1—H1B	109.5	C6—C1—H1A	120.1
C3—N1—H1C	109.5	C2—C1—H1A	120.1
H1B—N1—H1C	109.5	C7—C8—H8A	109.5
C3—N1—H1D	109.5	C7—C8—H8B	109.5
H1B—N1—H1D	109.5	H8A—C8—H8B	109.5
H1C—N1—H1D	109.5	C7—C8—H8C	109.5
O1—C6—C5	115.7 (6)	H8A—C8—H8C	109.5
O1—C6—C1	125.3 (6)	H8B—C8—H8C	109.5
C5—C6—C1	119.0 (6)	C3—C2—C1	120.0 (6)
C5—C4—C3	119.5 (5)	C3—C2—H2A	120.0
C5—C4—H4A	120.3	C1—C2—H2A	120.0
C3—C4—H4A	120.3	O1—C7—C8	107.8 (6)
C2—C3—C4	120.6 (6)	O1—C7—H7A	110.1
C2—C3—N1	120.8 (5)	C8—C7—H7A	110.1
C4—C3—N1	118.5 (5)	O1—C7—H7B	110.1
C4—C5—C6	121.1 (6)	C8—C7—H7B	110.1
C4—C5—H5A	119.5	H7A—C7—H7B	108.5
C6—C5—H5A	119.5		
			
C7—O1—C6—C5	173.5 (6)	O1—C6—C1—C2	178.9 (7)
C7—O1—C6—C1	−7.8 (10)	C5—C6—C1—C2	−2.6 (11)
C5—C4—C3—C2	0.3 (10)	C4—C3—C2—C1	−0.1 (10)
C5—C4—C3—N1	176.6 (6)	N1—C3—C2—C1	−176.3 (6)
C3—C4—C5—C6	−1.6 (10)	C6—C1—C2—C3	1.3 (11)
O1—C6—C5—C4	−178.6 (6)	C6—O1—C7—C8	−174.7 (6)
C1—C6—C5—C4	2.7 (11)		

### Hydrogen-bond geometry (Å, °)

**Table:**

*Cg*1 is the centroid of the benzene ring.

**Table:**

*D*—H···*A*	*D*—H	H···*A*	*D*···*A*	*D*—H···*A*
N1—H1B···Br1	0.89	2.78	3.368 (4)	125
N1—H1B···Br1^i^	0.89	2.76	3.324 (5)	122
N1—H1C···Br1^ii^	0.89	2.56	3.375 (4)	153
N1—H1D···Br1^iii^	0.89	2.51	3.348 (5)	158
C7—H7A···Cg1^iv^	0.97	3.01	3.674 (8)	127
C8—H8B···Cg1^v^	0.96	2.96	3.677 (8)	132

Symmetry codes: (i) −*x*, *y*−1/2, −*z*+1/2; (ii) *x*, *y*−1, *z*; (iii) −*x*, −*y*+1, −*z*; (iv) −*x*+1, *y*+1/2, −*z*+1/2; (v) −*x*+1, −*y*+1, −*z*.
